# Individual differences in stress coping are linked to working memory performance in male and female F344 rats

**DOI:** 10.1016/j.physbeh.2025.115101

**Published:** 2025-09-09

**Authors:** Melanie A. Tieman, Hannah M. Gandy, Haley A. Dufala, Caitlin A. Orsini, Lori A. Newman, Joseph A. McQuail

**Affiliations:** a Department of Pharmacology, Physiology, and Neuroscience, University of South Carolina School of Medicine, Columbia, SC, USA; b Department of Psychology, University of Texas at Austin, Austin TX, USA; c Department of Psychological Science, Vassar College, Poughkeepsie, NY, USA

**Keywords:** Working memory, stress coping, Sex differences, Corticosterone, Prefrontal cortex, Behavioral phenotyping, Individual differences

## Abstract

Executive dysfunction and altered stress reactivity are core features of many neuropsychiatric disorders, contributing substantially to global disability and frequently exhibiting sex-specific prevalence and symptom profiles. Understanding how stress responses normally relate to executive function, and whether these relationships differ by sex, is critical for identifying mechanisms of vulnerability and resilience. Although sex differences in stress and cognition have been reported, preclinical findings remain mixed, and few studies assess behavioral coping, endocrine responses, and working memory performance within the same subjects. In this study, we used F344 rats to examine how individual differences in behavioral and hormonal responses to acute stress relate to working memory, assessed using an operant delayed match-to-sample task. Males and females differed in stress reactivity: females exhibited greater immobility across “pre-test” and “test” sessions of the forced swim test (FST) and a more rapid decline in corticosterone (CORT) following restraint, suggesting sex-specific patterns of behavioral and physiological regulation. Despite these differences, working memory accuracy did not differ by sex, although females completed fewer trials and responded more slowly than males. Estrous cycle did not influence cognitive or non-mnemonic performance. Crucially, individual variation in FST immobility, rather than sex or CORT dynamics, was the strongest predictor of working memory accuracy. These findings bridge observed sex differences in stress reactivity with the absence of sex differences in cognition, revealing a trait-level relationship that generalizes across sexes. By jointly considering sex and individual behavioral traits, this work supports approaches to identify biologically meaningful cognitive phenotypes in preclinical models.

## 1. Introduction

Neuropsychiatric disorders, including major depressive disorder (MDD), schizophrenia (SCZ), post-traumatic stress disorder (PTSD), anxiety, and attention-deficit/hyperactivity disorder (ADHD), represent a major public health challenge, affecting hundreds of millions of individuals globally and ranking among the leading causes of disability [1, 2]. These conditions frequently emerge early in life and may persist across the lifespan, with nearly half of the global population projected to experience at least one diagnosable mental health condition by age 75 [2]. Critically, these disorders exhibit consistent and clinically meaningful sex differences: MDD and anxiety are more prevalent in females; SCZ and ADHD are more common in males; and PTSD is both more prevalent and symptomatically distinct in women [2,3]. Across diagnostic categories, impairments in cognitive control and stress regulation, both supported by the prefrontal cortex (PFC), are common, suggesting shared neurobiological mechanisms that cut across traditional diagnoses [4]. These transdiagnostic impairments impact well-being and functional capacity, while also complicating diagnosis, treatment, and prevention. As such, they underscore the need for public health strategies that address sex-associated differences in risk and highlight stress-related cognitive dysfunction as a central contributor to the development and course of mental illness.

The neurobiological systems that support cognitive control and stress regulation are deeply interconnected and contribute to distinct behavioral and physiological profiles across sexes. Working memory, a core executive function supported by the PFC, is essential for goal-directed behavior and particularly sensitive to stress in both humans [5,6] and rodents [7,8]. The hypothalamic-pituitary-adrenal (HPA) axis coordinates the physiological stress response via glucocorticoid release, which in turn modulates PFC activity through feedback mechanisms [9, 10]. Rodent behavioral studies have been instrumental in elucidating sex differences in PFC-dependent working memory and stress reactivity. For example, male rats tend to outperform females in spatial working memory tasks such as the radial arm maze, though this advantage is not universally observed [11] and may reflect estrogenic modulation of dorsal hippocampal and striatal circuits, which can bias females toward or away from spatially guided strategies [12–14]. Furthermore, acute stress that impairs spatial working memory of male rats can enhance such cognition in females, even in the presence of, and without interacting with, expected effects of the estrous cycle [15]. While these findings have traditionally been interpreted through the lens of group-level sex comparisons, emerging perspectives highlight the importance of accounting for within-group variability in cognitive and stress-related outcomes. Meta-analyses demonstrate that individual differences, shaped by factors such as stressor type, task demands, timing, and hormonal milieu, account for substantial variability in working memory and stress reactivity, often exceeding the explanatory power of sex alone [6,16].

Emphasizing individual variability, both within and across sexes, reflects a broader shift toward dimensional, mechanism-focused approaches. Rodent studies offer a tractable system for experimentally probing how behavioral, physiological, and neural processes influence cognition and stress responses, insights that can inform hypotheses relevant to human health. Working memory can be robustly assessed using operant tasks like delayed match-to-sample (DMTS), which tax PFC-dependent processes by requiring maintenance and updating of information across variable delays [17]. Coping styles, often characterized as active or passive, capture trait-like differences in stress appraisal and are frequently assessed using the forced swim test (FST), where immobility is interpreted as a passive strategy that varies reliably across individuals [18,19]. Glucocorticoid dynamics offer a physiological complement to behavioral phenotyping, with peak hormone levels and recovery slopes indexing the efficiency of HPA axis activation and negative feedback [9,10]. Treating these behavioral and endocrine measures as continuous traits enables a more nuanced understanding of how stress and cognition interact across individuals. This dimensional approach aligns with a growing emphasis in neuroscience on cross-domain integration, translational relevance, and identification of conserved mechanisms of vulnerability and resilience, principles central to precision medicine and dimensional psychiatry frameworks [20,21].

Although stress and cognition are frequently studied together, few investigations have concurrently assessed working memory, coping behavior, and glucocorticoid regulation within the same subjects. This gap limits the ability to evaluate how individual differences in stress reactivity correspond with executive function. Often, sex is treated as a binary variable despite evidence that substantial within-sex variability exists, and that trait-level differences, such as coping style or hormone regulation, may better explain cognitive outcomes than sex alone [11, 18,22]. To overcome these limitations, the present study integrates cognitive, behavioral, and endocrine measures within a sex-inclusive preclinical model. By employing the DMTS task, FST, and repeated corticosterone (CORT) assays, this design captures variation in working memory, stress coping, and glucocorticoid regulation among individual rats. Males and females were equally represented to ensure observed patterns encompass the full spectrum of biological and behavioral variability. Although sex is commonly treated as a categorical grouping variable in preclinical research, this study additionally analyzes individual variability both within and across sexes. Rather than focusing solely on sex-based group comparisons, we adopted an exploratory, trait-based approach to examine how coping behavior and physiological stress responses relate to PFC-dependent cognitive performance. This strategy aims to identify biologically meaningful patterns that may inform future models of cognitive resilience and vulnerability, aligning with emerging frameworks in translational neuroscience that emphasize dimensional analysis and mechanistic integration.

## 2. Materials and methods

### 2.1. Subjects

All procedures conducted with live animals were reviewed and approved by the University of South Carolina’s Institutional Animal Care and Use Committee and conformed to the National Institutes of Health Guide for the Care and Use of Laboratory Animals. Male and female (*n* = 11/sex) F344 (CDF) rats were purchased at 60 days of age from Charles River Laboratories (Raleigh, NC, USA). We selected the F344 strain because we have previously evaluated relevant behavioral and neuroendocrine parameters of male F344 rats and drew upon those data to inform the design of the present work [23,24]. Animals used in this study were housed in the Animal Resource Facility at the University of South Carolina School of Medicine – Columbia. The housing room was maintained within a set temperature range (20–25 °C) and on a regular 12:12 h light:dark cycle (lights on at 0700 h). Each rat was housed individually in plastic cages (25.5 × 35.75 × 18 cm) lined with Sani--Chip^®^ bedding (Teklad 7090; Envigo, Indianapolis, IN, USA). Access to chow (Teklad Diet 8604) was restricted throughout operant testing to motivate task performance. During this phase of the study, rats were weighed immediately after testing and fed sufficient chow to maintain 85 % of free-feeding body weight, as determined at the start of operant testing and after adjusting for expected growth. Rats were restored to ad libitum food access after conclusion of operant testing. Water was freely available in the home-cage during all phases of these experiments and access was only intermittently interrupted for the minimum time needed to accomplish behavioral procedures outside of the home-cage.

### 2.2. Operant testing apparatus

Operant shaping and testing procedures were performed in eight HABITEST chambers (30.5 × 25.4 × 30.5 cm, Coulbourn Instruments, Whitehall, PA, USA) enclosed within sound attenuating cubicles. The front and back walls of the HABITEST chamber were constructed from Plexiglas. One side wall was constructed from metal and outfitted with retractable levers (H23–17RC, Coulbourn Instruments) mounted to either side of a recessed trough (H14–01R) into which food pellet rewards (TestDiet 1811,155 (5TUL), LabTab^™^ AIN-76A Rodent 45 mg; Lab Supply, Fort Worth, TX, USA) were dispensed from a food hopper (H14–23R, Coulbourn Instruments). This trough was also fitted with a photobeam (H20–94, Coulbourn Instruments) to detect head entries and a 1.12 W lamp for illumination. The opposite wall was constructed of metal and contained a 1.12 W house light. All stimulus-response modules were wired into circuit boards that connected to a computer to control experiments and record responses via Graphic State 4 software (Coulbourn Instruments).

### 2.3. Operant shaping procedures

Operant procedures were conducted between 0900–1300 h daily. In the first stage of shaping, 38 pellets were dispensed at a rate of one pellet every 100 ± 40 s over 64 min. In the second stage of testing, a single lever (left or right, counterbalanced across boxes) was extended and lever presses were reinforced with delivery of a single pellet (FR1). After reaching a criterion of 50 lever presses in 30 min, rats were then trained on the opposite lever using the same procedures. In the third stage of shaping, the left or right lever (counterbalanced across trials) was extended into the chamber and each press resulted in a single food pellet delivery (FR1) until achieving 80 lever presses in a 30-minute session. In the fourth stage of shaping, the left or right lever (counterbalanced across trials) was extended into the chamber, and two successive presses were required to receive a single food pellet (FR2). Rats were required to complete at least 80 lever presses in a 30-minute session to move to the next phase of shaping.

In the final stage of shaping, rats learned to apply a “matching” rule, with correct responses reinforced by food rewards. Each trial began with the presentation of a single lever, referred to as the sample lever, randomly positioned on the left or right side of the chamber. After the rat pressed the sample lever, it was retracted, and the food trough light was illuminated. A nose-poke at the food trough immediately triggered the choice phase, during which both levers were extended. If the rat pressed the same lever that had been presented during the sample phase, this was considered a correct “matching” response, and both levers retracted before a single food pellet was dispensed. Entry into the food trough to collect the pellet initiated a 5-second intertrial interval, after which the next trial began. If the rat instead pressed the opposite lever, this was considered an incorrect “non-matching” response. In that case, both levers retracted, and the house light was extinguished for a 5-second timeout period. After the timeout, a correction trial was presented in which the same sample lever was repeated. If the rat again made a non-matching error, the next trial was a forced choice in which only the correct lever was presented during the choice phase to prevent development of a side bias. Shaping was considered complete once rats reached a criterion of 80 % correct choices and no fewer than 70 completed trials (excluding forced choice trials) for two consecutive sessions.

### 2.4. Delayed match-to-sample testing

The operant task is adapted from Sloan et al. [17] and has been used by our lab previously to evaluate medial PFC-dependent cognition in F344 rats [23,24]. Each DMTS trial was comprised of three phases. During the “sample” phase, the left/right position of the sample lever was randomized within each pair of trials. A lever press caused the sample lever to retract and initiated the delay period (0, 2, 4, 8, 12, 18, and 24 s; order of delays was randomized within each block of seven trials) during which time rats were required to nose-poke into the central food trough. The first nose-poke following the programed delay interval initiated the “choice” phase of the task in which both levers extended into the chamber. If the rat pressed the lever that was presented during the sample phase (a correct response), both levers were retracted, and a single food pellet was delivered. Entry into the food trough to collect the food pellet initiated a 5-second intertrial interval, after which the next trial was initiated. If the rat pressed the opposite lever from the one presented during the sample phase (an incorrect response), both levers were retracted, and the rat received a 5-second “timeout” period during which the house light was extinguished. The next trial began immediately after the timeout period. Critically, progression through each phase required a specific response (lever press or nose-poke) with no imposed time limit. This design minimized confounds from trial omissions at longer delays—where accuracy typically declines—by ensuring that all rats completed comparable numbers of trials across delays. Rats were tested in one 40-minute session per day for 42 days, providing a sufficiently long window to capture both acquisition and stable performance. Stability was not assumed in advance but was confirmed through retrospective analysis of performance across the testing period. The primary outcome of interest was the number of correct and incorrect choices at each delay. Secondary metrics included latency to press levers during the sample and choice phases of each trial.

### 2.5. Vaginal lavage and cytology to determine estrous stage during delayed match-to-sample testing

A subset of females (*n* = 6) continued testing on the DMTS task beyond the initial 42-session period, during which estrous cycle stage was monitored concurrently. This follow-up phase was added after the primary dataset had been collected to assess whether hormonal fluctuations across the estrous cycle influenced working memory or non-mnemonic performance. After the conclusion of daily DMTS testing, each female was gently restrained while 200 μl of warmed (37 °C) 0.9 % saline was washed into the vaginal canal to collect cells for analysis. Lavages were deposited onto glass microscope slides and permitted to dry fully before staining with Toluidine blue (AC348600250; ACROS Organics, Somerville, NJ, USA). After cover slipping with Permount (SP15–100; Fisher Scientific, Atlanta, GA, USA), stained cells were viewed with a light microscope and classified as characteristic of proestrus, estrus, metestrus or diestrus according to the criteria of Cora et al. [25].

### 2.6. Behavioral and hormonal responses to acute stress

After the conclusion of operant testing, rats were allowed to rest for at least one week prior to FST or physical restraint. The order in which stressors were presented was counterbalanced; one-half of the study sample underwent FST first, which was followed by physical restraint one week later. The other half of the study sample was exposed to the stressors in the reverse order.

### 2.7. Forced swim test

FST was conducted according to the methods of Slattery & Cryan [26]. All testing was performed between 1300–1500 h and conducted in one of four identical, transparent Plexiglas cylinders (height: 61 cm; diameter: 30 cm) filled to a depth of 45 cm with 24 ± 1 °C water. Cylinders were enclosed on three sides by black walls to provide a contrasting background against which activity was recorded via digital camera. Each rat received a 900 s “pre-test” session followed by a 300 s “test” session 24 h later. Rats were thoroughly dried and returned to centralized housing after recovery from each testing session. Video recordings were subsequently viewed using VLC media player (version 3.0.11; VideoLAN, Paris, France) and immobility, swimming, climbing, and diving behaviors were scored continuously using Kinoscope software (version 0.3.0.4, [27]). The duration of immobility in the pre-test and test sessions was the chief metric of interest.

### 2.8. Physical restraint, blood collection, and corticosterone measurement

Between 0900–1100 h, rats were immobilized in wire-mesh restrainers for 60 min in the home-cage. Blood was collected from the lateral tail vein into Microvette^®^ CB 300 K2EDTA-coated capillary tubes (16.444.100; Sarstedt, Newton, NC, USA) at 0, 60, 120, 180 and 300 min after the onset of restraint. Whole blood was centrifuged at 3000 g for 10 min at +4 °C; plasma was retained and stored at −80 °C. Plasma CORT concentration was assayed in duplicate using an ELISA kit (ADI-901–097; ENZO Life Sciences, Farmingdale, NY, USA) according to the manufacturer’s protocol. Absorbance at 405 nm was measured using a Synergy 2.0 plate reader (BioTek, Winooski, VT, USA) connected to a computer running Gen5 software (version 1.05, BioTek). Every plate included standardized reactions prepared from known concentrations of CORT (32–20,000 pg/ml). For each standard curve, log-transformed data were fitted to a non-linear, variable slope equation using Prism software (version 8; GraphPad Software, San Diego, CA, USA) and the goodness of fit (r2) for all experiments was 0.998 or greater. The concentration of unknown samples was determined by interpolating raw absorbances to the standard curve.

### 2.9. Statistical analyses

For DMTS task performance, the primary outcome was choice accuracy, defined as the percentage of correct responses at each delay. As a secondary, non-cognitive measure, we also calculated total trials completed, defined as the sum of correct and incorrect choices within each session. To assess changes in performance over time, choice accuracy was averaged across consecutive sets of seven daily sessions, referred to here as weeks of testing (Week 1–6). These data were analyzed using a mixed-factor ANOVA with sex (2 levels: male, female) as a between-subjects factor and both testing week (6 levels) and delay (7 levels: 0, 2, 4, 8, 12, 18, and 24 s) as within-subjects factors. In this and all subsequent comparisons, *p* < 0.05 was considered statistically significant. The Huynh–Feldt correction was applied to all repeated-measures data, and Holm-adjusted post hoc comparisons were used to evaluate main effects. Stable performance was defined retrospectively as the absence of statistically significant differences in choice accuracy across successive weeks of testing (e.g., Week 4 vs. Week 5, Week 5 vs. Week 6). Data from weeks meeting this criterion were averaged and reanalyzed using a sex-by-delay ANOVA. Additional comparisons between sexes were conducted using independent samples *t*-tests for total trials completed, sample latency, and correct choice latency.

Variance of choice accuracy in relation to estrous cycle was analyzed using a two-factor repeated-measures ANOVA, with estrous phase (3 levels: proestrus, estrus, and diestrus) and delay as the repeated factors. A one-factor ANOVA was used to test the effect of estrous stage on total trials completed in each session. Metestrus was not consistently observed in all rats, and therefore, not included in our analysis.

Duration of immobility (in seconds) during the 900-second FST pre-test was analyzed using a mixed factors ANOVA, with sex as a between-subjects factor and time bin as a repeated factor (3 levels: 0–300, 300–600, 600–900 s). An independent samples *t*-test was used to compare duration of immobility during the 300-second FST test between males and females.

Plasma concentration of CORT was analyzed using a two-factor ANOVA, with sex as a between-subjects factor and timepoint (5 levels: 0, 60, 120, 180, and 300 min) as a repeated factor. To assess individual differences in stress reactivity and recovery, total CORT exposure was quantified as area under the curve (AUC), log-transformed using the natural logarithm (ln[CORT AUC]) to improve normality and compared between sexes using independent-samples *t*-tests. Additionally, slopes were calculated across defined intervals (0–60 and 60–120 min postonset) and tested for sex differences using the same approach.

To evaluate the relationship between individual differences in stress-related responses and working memory performance, we conducted multiple linear regression analyses using summary metrics from each behavioral and endocrine assay. Working memory performance was indexed by DMTS choice accuracy, calculated as the percentage of correct responses across a subset of sessions during which performance had stabilized. The identification of this stable performance window was based on retrospective analysis described in the Results section. Predictor variables included total immobility duration during the 900-second pre-test and 300-second test sessions of the FST, the slope of CORT rise during restraint (0–60 min), the slope of CORT decline during early recovery (60–120 min), and total CORT exposure (ln[CORT AUC]). Two regression models were fit: the first included only the behavioral and hormonal predictors, and the second included biological sex as an additional covariate to evaluate whether sex accounted for variance in working memory performance or moderated any observed relationships.

## 3. Results

### 3.1. Working memory choice accuracy is not different between sexes

**Fig. 1.**
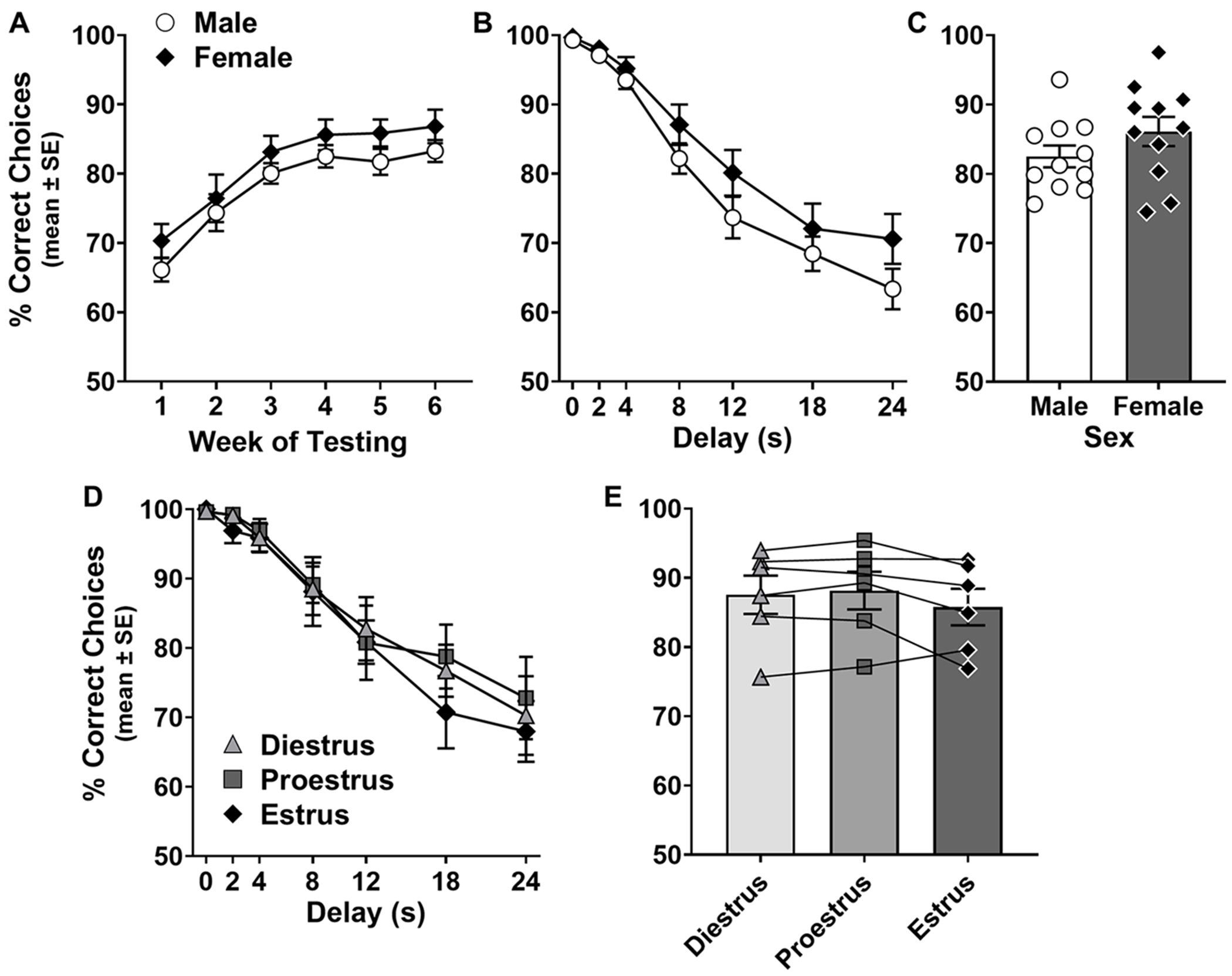
Working memory is similar between male and female rats and across phases of the estrous cycle. **A:** Choice accuracy (% correct choices; y-axis) improved significantly across early weeks of testing (1–3) and stabilized at asymptotic levels in later weeks (4–6), with no significant differences between males (white circles) and females (black diamonds) across weeks (x-axis). **B & C:** When averaged across stable weeks of testing (4–6), working memory accuracy declined as a function of increasing programmed delay intervals (0–24 s; x-axis) between the sample and choice phases. Delay-dependent declines were observed in both sexes and did not interact with sex, indicating that working memory performance was highly stable and comparable between male and female rats. **D & E:** Choice accuracy declined with increasing delay intervals across phases of the estrous cycle in female rats, with no significant differences in overall accuracy or delay sensitivity between phases, indicating stable working memory performance across hormonal states.

There were no reliable differences in choice accuracy between male and female rats (Fig. 1; (1, 20) = 1.988, *p* = 0.174, η^2^*p* = 0.090). The main effect of delay was significant, indicating that choice accuracy decreased with longer delays (F(1.672, 33.441) = 235.152, *p* < 0.001, η^2^*p* = 0.922). The main effect of testing week was also significant, indicating changes in choice accuracy across time (F(1.932, 38.636) = 35.579, *p <* 0.001, η^2^*p* = 0.640). The interaction between delay and sex was not significant, suggesting the effect of delay on choice accuracy did not differ between male and female rats (F(1.672, 33.441) = 1.265, *p* = 0.290, η^2^*p* = 0.059). Similarly, the interaction between performance week and sex was not significant, indicating similar training effects in both sexes over time (F(1.932, 38.636) = 0.129, *p* = 0.872, η^2^*p* = 0.006). There was a significant interaction between week and delay, suggesting the effect of delay on choice accuracy varied over the course of training (F(30, 600) = 4.017, *p* < 0.001, η^2^*p* = 0.167). The interaction between performance week, delay and sex, however, was not significant (F(30, 600) = 0.680, *p* = 0.902, η^2^*p* = 0.033). Considered together, these results suggest that over the course of training in the task, choice accuracy improved to the same extent in males and females.

Post hoc comparisons were conducted to examine changes in choice accuracy across weeks of testing (Fig. 1A). The comparison between Week 1 and Week 2 revealed a significant increase in choice accuracy (Mean Difference = 7.194, *t* = 5.896, pHolm < 0.001, Cohen’s *d* = 0.747). Similarly, Week 2 and Week 3 showed a significant improvement in choice accuracy (Mean Difference = 6.179, *t* = 3.18, pHolm = 0.028, Cohen’s *d* = 0.642). The comparison between Week 3 and Week 4 indicated a significant increase in choice accuracy (Mean Difference = 2.47, *t* = 3.061, pHolm = 0.031, Cohen’s *d* = 0.257). However, the difference between Week 4 and Week 5 was not significant (Mean Difference = −0.272, *t* = −0.336, pHolm = 0.74, Cohen’s *d* = −0.028). Lastly, the comparison between Week 5 and Week 6 did not show a significant difference in choice accuracy (Mean Difference = −1.256, *t* = −1.59, pHolm = 0.383, Cohen’s *d* = −0.13).

Given that performance improved significantly in a stepwise fashion through Week 4 and remained stable thereafter, performance across Weeks 4, 5, and 6 was collapsed and re-tested for effects of sex and delay (Fig. 1B). Consistent with the results of the parent ANOVA, the main effect of sex on choice accuracy was not significant, indicating that males and females performed similarly on this measure (F(1, 20) = 1.859, *p* = 0.188, η^2^*p* = 0.085; Fig. 1C). Also consistent with the parent ANOVA, there was still a main effect of delay, with a decrease in choice accuracy at longer delays (F(1.844, 36.877) = 137.769, *p* < 0.001, η^2^*p* = 0.873), and this behavioral pattern did not differ between sexes F(1.844, 36.877) = 1.443, *p* = 0.249, η^2^*p* = 0.067). Post hoc comparisons for the delay conditions confirmed significant decreases in choice accuracy with increasing delay intervals. Specifically, choice accuracy significantly decreased from 0 to 2 s (Mean Difference = −1.94, *t* = −4.665, pHolm < 0.001, Cohen’s *d* = −0.246), 2 to 4 s (Mean Difference = −3.193, *t* = −4.209, pHolm < 0.001, Cohen’s *d* = −0.404), 4 to 8 s (Mean Difference = −9.716, *t* = −8.847, pHolm < 0.001, Cohen’s *d* = −1.23), 8 to 12 s (Mean Difference = −7.725, *t* = −8.844, pHolm < 0.001, Cohen’s *d* = −0.978), 12 to 18 s (Mean Difference = −6.655, *t* = −5.749, pHolm < 0.001, Cohen’s *d* = −0.843), and 18 to 24 s (Mean Difference = −3.276, *t* = −5.348, pHolm < 0.001, Cohen’s *d* = −0.415). These reliable effects confirm that working memory was consistently taxed with increasing delays, even after male and female rats had reached stable, asymptotic performance.

Non-mnemonic performance variables were compared between males and females in Weeks 4, 5, and 6 (Table 1). Females completed significantly fewer trials (t(20) = −2.217, *p* = 0.038, Cohen’s *d* = −0.945; Mean = 93.87, SD = 25.592) compared with males (Mean = 113.317, SD = 13.845). The Mann-Whitney U test was used to analyze latency to press the sample lever due to the deviation from normality in the male group (*W* = 0.599, *p* < 0.001). The test did not indicate a significant difference between males and females (*U* = 88, *p* = 0.076, rank biserial correlation = 0.455), although there was a trend toward a main effect of sex, with females taking longer to press the sample lever (Mean = 2.15 s, SD = 0.951) relative to males (Mean = 1.656 s, SD = 0.996). The Mann-Whitney U test was also used to analyze latency to press the sample (i.e., correct) lever during the choice phase due to the deviation from normality in the female group (*W* = 0.844, *p* = 0.035). There was a significant difference between males and females (*U* = 94, *p* = 0.028, rank biserial correlation = 0.554), with females (Mean = 2.18 s, SD = 1.041) taking longer to press the lever than males (Mean = 1.378 s, SD = 0.288). The fewer number of completed trials in females in the absence of sex differences in overall choice accuracy is likely attributable to their longer response times to respond on the correct lever in the choice phase and to initiate the next trial by responding on the sample lever in the sample phase.

### 3.2. Working memory accuracy of females does not fluctuate with estrous cycle

The frequency of observed proestrus (27 % of smears), estrus (15 %), and diestrus (54 %), as determined by cytological evaluation of vaginal smears obtained after daily testing, approximated typical differences in the duration of each estrous phase in the rat (Cora et al., 2015). Metestrus, which lasts only 6–8 h in rats, was not frequently observed (5 % of smears), and was only ever observed in 3 out of the 6 females examined in this sub-study. Consequently, data from days when rats were determined to be in metestrus were excluded from analysis.

A repeated-measures ANOVA examining the effects of estrous phase and delay on choice accuracy revealed no significant differences in choice accuracy across the different phases of the estrous cycle (F(1.253, 6.265) = 2.065, *p* = 0.202, η^2^*p* = 0.292), nor was there any reliable interaction with delay (F(12, 60) = 1.132, *p* = 0.352, η^2^*p* = 0.185; Fig. 1D & E). However, the main effect of delay was significant (F(6, 30) = 28.363, *p* < 0.001, η^2^*p* = 0.85), indicating that choice accuracy decreased with longer delays. Further analysis of non-mnemonic performance measures, including the number of total completed trials, sample latency, and correct choice latency, revealed no significant differences in any variable across the phases of the estrous cycle [Table 2; completed trials: F(2, 10) = 0.341, *p* = 0.719, η^2^*p* = 0.064; sample latency: F(2, 10) = 1.001, *p* = 0.402, η^2^*p* = 0.167; correct choice latency: F (2, 10) = 0.775, *p* = 0.486, η^2^*p* = 0.134]. These findings suggest that the phase of the estrous cycle does not significantly impact non-mnemonic aspects of task performance.

### 3.3. Females exhibit greater immobility than males across all phases of the forced swim test

**Fig. 2.**
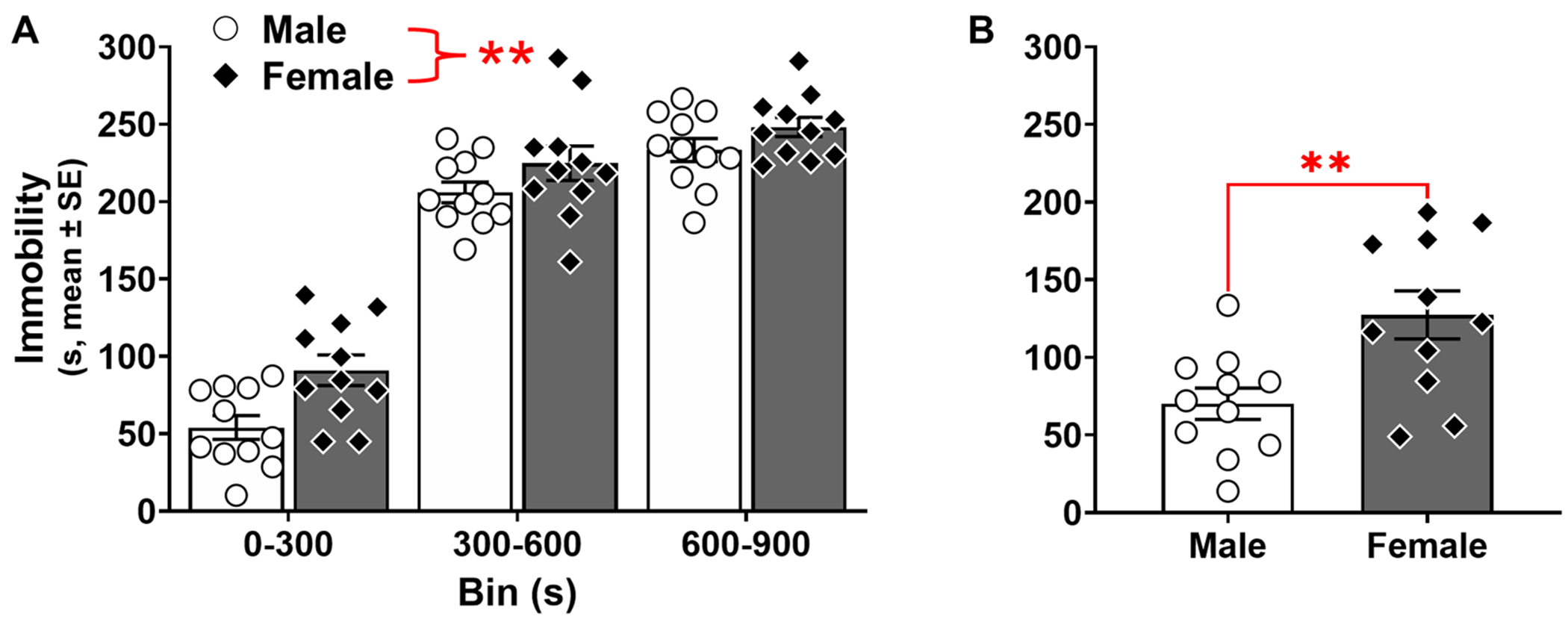
Female rats exhibit greater immobility than males during pre-test and test phases of the forced swim test. **A:** Immobility duration (y-axis) during the 900-second pre-test was analyzed in three 300-second time bins (x-axis). Females (black diamonds) were significantly more immobile than males (white circles) across all bins, indicating greater passive coping behavior. Immobility increased significantly across time bins in both sexes, with no sex × time interaction, suggesting a similar temporal pattern of behavioral adaptation. **B:** During the 300-second test session conducted 24 h later, females again exhibited significantly greater total immobility than males, consistent with enhanced passive coping across both sessions. Asterisks indicate significant main effects of sex (**p < 0.01).

The analysis of immobility during the 900-second forced swim pre-test, separated into three 300-second time bins, revealed significant differences between male and female rats (Fig. 2A). Females exhibited longer durations of immobility than males (F(1, 20) = 9.625, *p* = 0.006, η^2^*p* = 0.325), with post hoc comparisons confirming that females were significantly more immobile (*t* = 3.102, pHolm = 0.006, Cohen’s *d* = 0.851). The main effect of bin was significant (F(1.925, 38.508) = 269.243, *p* < 0.001, η^2^*p* = 0.931), and stepwise comparisons showed significant increases in immobility from 0 to 300 s to 300–600 s (Mean Difference = 142.814, *t* = −15.656, pHolm < 0.001, Cohen’s *d* = −5.165) and from 300 to 600 s to 600–900 s (Mean Difference = 25.398, *t* = −3.639, pHolm = 0.002, Cohen’s *d* = −0.919). The interaction between bin and sex was not significant, suggesting the pattern of increasing immobility over time was similar across sexes (F(1.925, 38.508) = 1.139, *p* = 0.329, η^2^*p* = 0.054). These findings highlight the significant impact of sex on immobility, with females showing greater passive coping behavior, and confirm the progressive increase in immobility across the time bins during the forced swim pre-test in both sexes.

An independent samples *t*-test was conducted to compare immobility between female and male rats twenty-four hours later during the 300-second test phase of the forced swim. These analyses revealed a significantly greater duration of immobility in females relative to males (t(20) = 3.105, *p* = 0.006, Cohen’s *d* = 1.324; Fig. 2B).

### 3.4. Dynamics of corticosterone secretion differ between male and female rats during early recovery from physical restraint

**Fig. 3.**
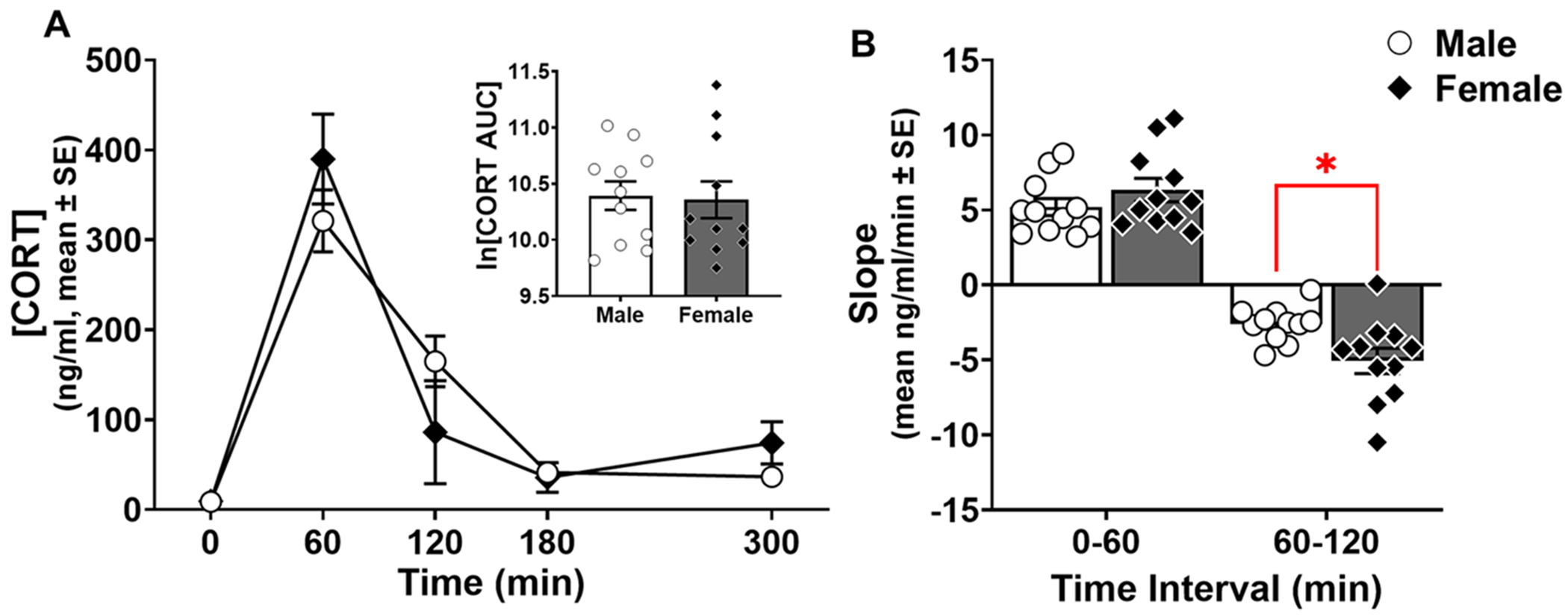
Dynamics of corticosterone secretion differ between male and female rats during early recovery from restraint stress. **A:** Plasma corticosterone concentration ([CORT]; y-axis) was measured at baseline and at 60, 120, 180, and 300 min following the onset of 60 min of physical restraint (x-axis). CORT levels increased significantly during restraint and declined during recovery in both males (white circles) and females (black diamonds). While no significant sex differences were observed at individual timepoints, the interaction between sex and timepoint was marginal (*p* = 0.097), suggesting subtle differences in the temporal dynamics of CORT regulation. Inset: Log-transformed area under the curve (ln[CORT AUC]) comparing the integrate CORT exposure during and following recovery from physical restraint. **B:** Slope values (y-axis) representing the rate of CORT change were calculated for two intervals: 0–60 min (restraint period) and 60–120 min (early recovery; x-axis). Females exhibited a significantly steeper decline in CORT during early recovery compared to males, indicating more rapid HPA axis feedback. Asterisks indicate significant sex difference (*p < 0.05).

A repeated measures ANOVA was conducted to analyze the effects of sex and timepoint on plasma CORT concentration before and after 60 min of physical restraint. There was no main effect of sex on plasma CORT concentration (F(1, 20) = 0.029, *p* = 0.866, η^2^*p* = 0.001; Fig. 3A), and this effect was confirmed by the absence of an effect of sex on ln (CORT AUC) (t(20) = −0.174, *p* = 0.867, Cohen’s *d* = −0.074; Fig 3A **inset**). Although there was a main effect of timepoint on plasma CORT concentration (F(2.282, 45.634) = 60.966, *p* < 0.001, η^2^*p* = 0.753), there was only a marginal interaction between timepoint and sex (F (2.282, 45.634) = 2.384, *p* = 0.097, η^2^*p* = 0.107), indicating that the change in CORT concentration occurred to a similar extent in both sexes. Stepwise post hoc comparisons revealed a significant increase from 0 to 60 min (Mean Difference = 346.264, *t* = 11.943, Cohen’s *d* = 3.529, pHolm < 0.001) and significant decreases from 60 to 120 min (Mean Difference = −229.929, *t* = −8.388, Cohen’s *d* = −2.343, pHolm < 0.001) and from 120 min to 180 min (Mean Difference = −87.045, *t* = −2.744, Cohen’s *d* = −0.887, pHolm = 0.038). There was no difference between 180 and 300 min (Mean Difference = 16.946, *t* = 1.199, Cohen’s *d* = 0.173, pHolm = 0.244). Collectively, these results confirm the anticipated increases of [CORT] in response to physical restraint and their subsequent decreases during recovery.

Regarding the marginal interaction between sex and time, post hoc comparisons between males and females indicated no significant differences at any timepoint (*t* = −1.231 to 1.544, pHolm = 0.138 to 0.888). However, our planned comparisons of slope values revealed sex differences in the dynamics of CORT secretion and recovery (Fig. 3B). During the active restraint period (i.e., 0–60 min), slope values did not differ significantly by sex (t(20) = 1.177, *p* = 0.253, *d* = 0.502), although females exhibited a numerically steeper rise in CORT (6.34 ± 2.60 ng/ml/min) relative to males (5.20 ± 1.87 ng/ml/min). In contrast, during the period of early recovery (i.e., 60–120 min), females (−5.06 ± 2.79 ng/ml/min) showed a significantly steeper decline in CORT compared with males (−2.60 ± 1.17 ng/ml/min; t(20) = −2.691, *p* = 0.014, *d* = −1.147).

### 3.5. Individual differences in working memory choice accuracy are associated with immobility during forced swim

To investigate associations between stress-related factors and working memory performance, we conducted multiple linear regression analyses using working memory choice accuracy (Weeks 4–6, 0–24 s delays) as the dependent variable. Predictor variables included immobility during the 900-second forced swim pre-test and 300-second test sessions, as well as the total CORT exposure (ln[CORT AUC]) and slopes of CORT rise during restraint (0–60 min) and decline during early recovery (60–120 min). A second version of the model included sex as a covariate to assess its influence on the predictive relationship between stress responsivity and working memory performance. In both models, immobility during the forced swim pre-test emerged as the only significant predictor of working memory accuracy, such that greater pre-test immobility was associated with better working memory performance.

**Fig. 4.**
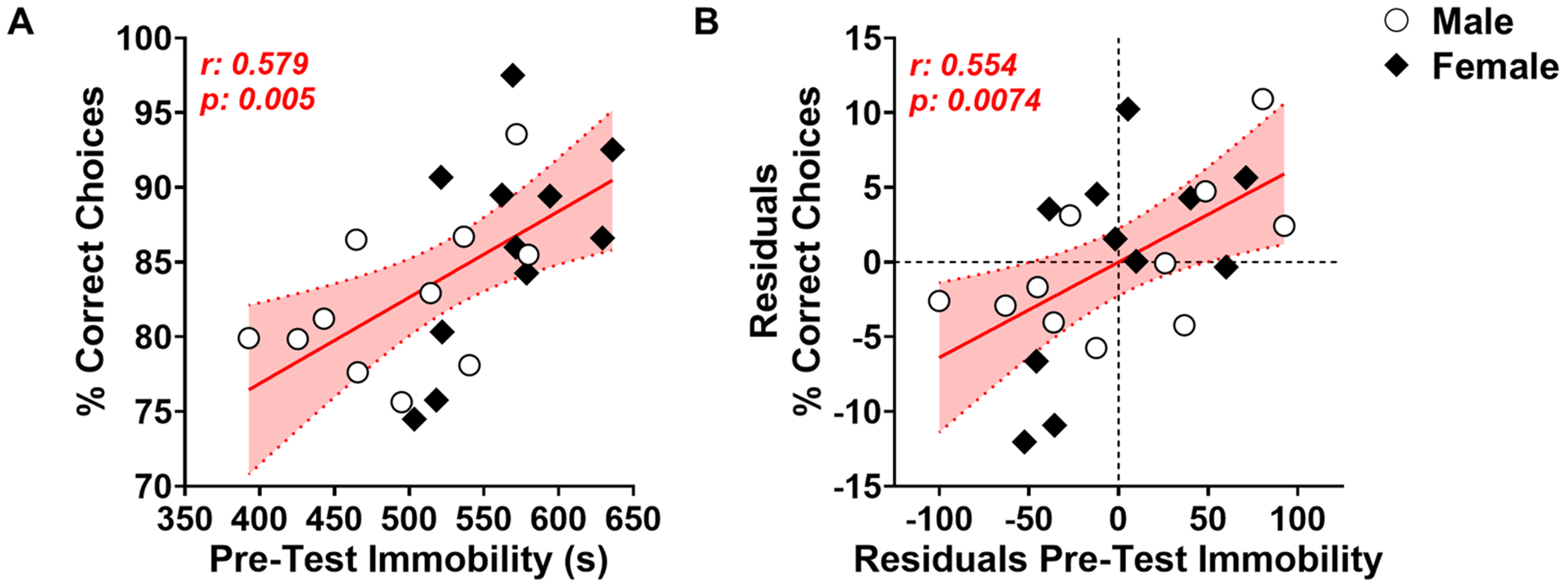
Individual differences in pre-test immobility predict working memory performance independent of biological sex and other stress-related measures. **A:** Scatterplot showing the bivariate relationship between working memory choice accuracy (y-axis; % correct choices averaged across Weeks 4–6, 0–24 s delays) and FST pre-test immobility duration (x-axis). Data points represent individual male (white circles) and female (black diamonds) rats. The fitted line represents the linear relationship between the raw values; corresponding r and p values are shown in the figure inset. **B:** Partial regression plot illustrating the unique association between working memory accuracy and FST pre-test immobility after adjusting for biological sex, FST test immobility, and CORT slope variables. Residuals of working memory accuracy (from the full model) are plotted against residuals of pre-test immobility. The fitted line reflects the significant linear relationship between these residuals; corresponding r and p values are shown in the figure inset. In the full model, pre-test immobility remained a significant predictor of working memory performance (β = 0.643, *p* = 0.021), while sex and other covariates were not significant.

Without sex in the model, pre-test immobility significantly predicted accuracy (*B* = 0.062, SE = 0.022, standardized β = 0.626, *t* = 2.793, *p* =0.013; Fig. 4A). The overall model was not statistically significant, but explained approximately 19 % of variance (adjusted R^2^ = 0.191, RMSE = 5.674; F(5, 16) = 1.991, *p* = 0.135). Neither immobility during the test phase (*p* = 0.969), the slope of CORT rise (*p* = 0.595), the slope of CORT decline (*p* = 0.683), nor ln(CORT AUC) (*p* = 0.819) contributed significantly to the prediction of working memory accuracy.

Including biological sex did not substantively change the relationship between pre-test immobility and working memory performance (*B* = 0.064, SE = 0.025, β = 0.643, *t* = 2.579, *p* = 0.021), and sex itself was not a significant predictor (*p* = 0.858; Fig. 4B). The overall model fit slightly decreased (adjusted R^2^ = 0.139, RMSE = 5.853; F(6, 15) = 1.564, *p* = 0.225). Consistent with the first model, immobility during the test phase (*p* = 0.925), the slope of CORT rise (*p* = 0.683), the slope of CORT decline (*p* = 0.686), and ln(CORT AUC) (*p* = 0.892) did not significantly predict working memory performance.

## 4. Discussion

Accurately identifying sex differences in preclinical models of PFC-dependent functions, such as cognition, stress-related coping strategies, and regulation of stress hormones, has important implications for translational research, particularly for neuropsychiatric disorders that disproportionately affect one sex. The present study demonstrates that stress coping style, rather than sex or endocrine measures, was the strongest predictor of PFC-dependent working memory performance in young adult F344 rats. Specifically, greater immobility during initial exposure to the FST was associated with higher accuracy on an operant DMTS task. This outcome is unexpected, as it challenges the prevailing assumption that active coping is inherently advantageous. Although females consistently displayed greater FST immobility, they did not differ from males in working memory accuracy or CORT secretion during restraint stress. The absence of a sex difference in CORT secretion is particularly notable given prior evidence of heightened stress reactivity in females, although this discrepancy may be attributable to the extensive behavioral testing history on a PFC-dependent working memory task prior to endocrine assessment. Collectively, these findings indicate that coping style provides a more robust predictor of PFC-dependent cognition than either sex or HPA axis activation.

### 4.1. Working memory in males and females

The study of sex differences in working memory is essential for understanding the biological and hormonal factors that influence cognitive performance. In rodents, particularly rats, sex differences in cognitive function are of considerable interest, as they may offer insight into how biological sex and sex hormones shape brain function and task performance. Our results showed that, despite some differences in non-mnemonic aspects of performance, such as trial completion and response latencies, working memory accuracy was similar across sexes. Both males and females demonstrated comparable task acquisition and were equally sensitive to brief delays that render memoranda vulnerable to interference or decay, a hallmark feature of working memory performance across species [17,28]. Further, we determined working memory accuracy did not fluctuate across phases of the estrous cycle in females. These findings suggest that, under the conditions tested, working memory performance in rats is not significantly influenced by biological sex or hormonal fluctuations, highlighting the robustness of this cognitive function across sexes.

In rodent models, evidence for male advantages in spatial working memory is modest and inconsistent. A meta-analysis reported a slight male advantage in radial arm maze performance, but most studies found no reliable sex differences, and none reported a female advantage [11]. Findings are further tempered by the absence of sex differences in other paradigms, such as the Morris water maze, and by mixed results in tasks requiring procedural learning [29–32]. Such variability may reflect differences in cognitive strategies, with males showing greater sensitivity to spatial location and females exhibiting stronger object recognition tendencies, though these effects are not consistently observed across tasks [33,34].

Interpretations of sex differences in PFC-dependent working memory are confounded when using tasks that also engage hippocampal or parahippocampal circuits, such as the radial arm maze, delayed alternation, delayed match-to-position, or object recognition [35–40]. Because estradiol modulates hippocampal and striatal circuits that can bias the use of spatial strategies during cognitive tasks [12–14], observed sex differences in these tasks may not reflect true PFC-dependent cognition. Although the DMTS task provides a rigorous measure of PFC-dependent working memory [17,41], findings remain inconsistent. Some studies report lower accuracy in females [42,43] whereas others, including the present work, find no sex difference [44]. Differences in how rats are introduced to DMTS may contribute to these mixed outcomes: some studies gradually increased retention intervals before full testing, others provided only brief exposure after shaping on 0-s delays, and still others trained rats extensively on other operant tasks before DMTS. By contrast, our design used continuous DMTS training with a full range of delays across six weeks, during which performance rose steadily and then stabilized. Notably, an analogous delayed response task in monkeys also revealed no sex differences [45], suggesting that PFC-dependent working memory is largely comparable between males and females across species.

Separate from measures of accuracy, females completed fewer trials and responded more slowly across trial phases in this self-paced working memory task. Similar sex differences in trial completion and response latencies have been reported previously [43,44]. One interpretation is that females are less motivated to engage in cognitively demanding tasks for food rewards, but this seems unlikely given the absence of sex differences in progressive ratio responding, a direct measure of motivational drive [44,46]. In fact, other work suggests the opposite pattern: females acquire operant tasks more quickly when negative reinforcement is used [47] and maintain demand for high-value rewards under increasing fixed ratio schedules [48,49]. Another possibility is a speed–accuracy trade-off as females were slower prior to selecting the correct lever, consistent with decision-making patterns across species [50]. However, this explanation is tempered by the observation that females were also slower to press the sample lever, a phase unrelated to cognitive demand or reward.

Overall, evidence for sex differences in rodent working memory is inconsistent and may depend on task, strategy, hormones, reinforcement type, and experience. Among studies that isolate PFC-dependent processes with delayed matching paradigms, including the present one, a modest majority suggest that males and females perform similarly in terms of accuracy, with the most reliable differences limited to slower response latencies and fewer completed trials in females. Whether these non-mnemonic effects reflect motivational factors, cognitive processing speed, or both, remains unresolved, underscoring the need for further work to clarify the mechanisms underlying sex-related variation in PFC-dependent cognition.

### 4.2. Sex differences in stress coping and HPA axis regulation

Sex differences in stress responses are shaped by interactions among neural circuits, hormones, and experiential history. In the present study, female F344 rats displayed greater immobility in the FST than males, consistent with prior reports that F344 females show more passive coping [51,52]. Across strains, sex differences in FST behavior are inconsistent [18], but the subset of studies using F344 rats point to a reliable female bias toward immobility, suggesting that this strain may reliably express sex-linked differences in coping style [19].

In contrast to behavior, our endocrine findings diverged from most of the F344 literature. Numerous studies have reported that female F344 rats exhibit higher restraint stress-induced CORT than males [51, 53–55]. In our cohort, however, there was no sex difference in peak CORT responses to restraint, and females instead showed a more rapid recovery during the 60–120-minute post-stress period. Such accelerated recovery in females is rarely observed in F344 rats and may reflect the influence of prior experimental history. Experience is known to shape HPA axis responsivity: neonatal and repeated handling produce long-term reductions in stress reactivity [56], while prolonged exposure to daily stressors or behavioral tasks leads to habituation of CORT responses [57]. These experience-dependent adaptations may therefore have masked or buffered the typical female elevation in stress-induced CORT observed in this strain.

The PFC provides a plausible mechanistic link between behavior and endocrine regulation as both a target of glucocorticoids and a regulator of HPA axis activity through descending inhibitory control. Lesion and Fos-mapping studies demonstrate its role in terminating stress-induced CORT secretion [9,10,58,59] and influencing coping styles during stress [60,61]. Notably, females show greater PFC activation following FST [62], and this enhanced PFC engagement could plausibly facilitate more efficient negative feedback, supporting their faster CORT recovery in certain experimental and experiential contexts.

Taken together, the F344 strain typically shows a female bias toward passive coping and, under most conditions, heightened stress-induced CORT. Our unexpected finding of equivalent peak responses, along with faster recovery in females, underscores the potential influence of behavioral history and methodological context on HPA axis outcomes. Extensive cognitive testing may itself modify stress responses relative to naïve rats, and this factor should be explicitly considered when designing experiments and interpreting results across studies.

### 4.3. Individual differences in stress coping and associations with working memory

In our study, individual differences in working memory performance were most reliably predicted by behavior during the initial FST exposure, specifically immobility during the pre-test (Day 1) session. Rats with greater immobility during the initial FST exposure exhibited higher choice accuracy on a DMTS working memory task. This association held across models with and without biological sex as a covariate, and sex itself was not a significant predictor. In contrast, stimulation of, and recovery from, restraint-induced CORT levels did not predict working memory performance, suggesting that behavioral responses to stress, rather than HPA axis activation or negative feedback, may more sensitively index cognitive outcomes under these conditions. These findings highlight the importance of inter-individual variation in stress responses and coping behaviors and suggest that initial stress appraisal and coping strategies may shape working memory function in meaningful ways.

Rodent models show acute stress can enhance PFC-dependent cognitive performance under certain conditions. Acute stressors such as forced swim, footshock, or restraint have been shown to improve behavioral performance on tasks requiring working memory and cognitive flexibility, including T-maze alternation, object recognition, and reversal learning [8,63–65]. These effects are supported by evidence that acute stress potentiates glutamatergic transmission in the PFC through glucocorticoid receptor-mediated pathways and enhances markers of synaptic plasticity such as BDNF and Arc [8,63,65,66]. Jointly, these behavioral and molecular data support the idea that acute stress, under certain conditions, is associated with enhancement, rather than impairment, of executive functions, including working memory.

Our observation that pre-test FST immobility predicts better working memory adds a novel behavioral correlate to this literature. Although FST immobility is frequently interpreted as behavioral despair, an alternative perspective views it as an adaptive, energy-conserving coping strategy [67]. From this perspective, greater immobility during initial stress exposure may index a more efficient or adaptive cognitive appraisal of the stressor, one that promotes disengagement from futile action and facilitates physiological states conducive to recovery or task re-engagement. This aligns with models proposing that the perceived control and salience of stressors critically shape subsequent cognitive and neuroendocrine outcomes. Importantly, our association with working memory was derived from the pre-test (Day 1) session of the FST, during which rats encounter an inescapable stressor for the first time. This timing likely captures intrinsic coping style or appraisal tendencies, rather than any learned behavior, distinguishing our findings from studies that assess immobility after repeated exposure.

Notably, while immobility during the FST predicted cognitive performance, changes in CORT levels during and after restraint stress did not. This null result should not be overinterpreted; however, the relationship between CORT responses and cognition may change with age. We previously reported that CORT secretion in response to 60 min of physical restraint was positively associated with individual differences in working memory of older (22-month-old) male F344 rats [23], an effect that mirrors positive associations among acute stress, cortisol, and working memory in older humans [68–70]. Critically, our study showed this association was driven by enhanced CORT responses in older rats with preserved working memory, that is, performance on par with young adults, whereas age-matched rats with impaired memory exhibited lower CORT responses, similar to those of younger animals. Thus, an age-dependent shift in the relationship between working memory and HPA axis reactivity may reflect a form of cognitive reserve, wherein molecular, cellular, and physiological adaptations help maintain cognitive performance despite age-related brain changes.

Our results suggest that individual differences in coping behavior during initial exposure to stress, specifically pre-test immobility in the FST, predict working memory performance in rats. This association was independent of sex and was not paralleled by stress-related adjustments to circulating CORT levels, underscoring the value of behavioral phenotyping in identifying cognitive resilience or vulnerability to stress. Our findings align with prior work showing stress-induced enhancements in working memory under specific conditions and extend this literature by identifying a behavioral predictor with potential translational relevance. Future work should further investigate how stress appraisal, coping strategies, and neuroendocrine responses interact to shape cognitive outcomes across individuals, species, and task demands.

## 5. Conclusion

This study advances our understanding of how individual behavioral traits, particularly coping responses to acute stress, can serve as meaningful predictors of cognitive performance, independent of sex or hormonal status. By integrating behavioral phenotyping with cognitive and endocrine assessments, we move beyond traditional group comparisons to uncover mechanisms that underlie variability in executive function. This approach enables a more nuanced view of cognitive resilience, one that accounts for the diversity of stress responses within and across sexes. Importantly, our findings suggest that behavioral markers, such as immobility during initial stress exposure, may offer a practical and scalable means of identifying cognitive subtypes in preclinical models. This opens new avenues for tailoring interventions to individual profiles, aligning with the goals of precision medicine. As the field continues to refine models of neuropsychiatric vulnerability, incorporating individual differences in stress appraisal and coping may prove essential for predicting cognitive outcomes and developing more targeted, effective treatments.

## Figures and Tables

**Table 1:** Summary of Non-Mnemonic Task Performance by Sex.

Measure	Male (Mean ± SD)	Female (Mean ± SD)	p-value
Total Trials	113.32 ± 13.85	93.87 ± 25.59	0.038
Sample Latency (s)	1.66 ± 1.00	2.15 ± 0.95	0.076
Correct Choice Latency (s)	1.38 ± 0.29	2.18 ± 1.04	0.028

**Table 2:** Summary of Non-Mnemonic Task Performance by Estrous Cycle Phase.

Measure	Diestrus (Mean ± SD)	Proestrus (Mean ± SD)	Estrus (Mean ± SD)	p-value
Total Trials	107.57 ± 10.37	110.33 ± 19.01	111.42 ± 14.76	0.719
Sample Latency (s)	1.60 ± 0.36	1.67 ± 0.38	1.68 ± 0.44	0.402
Correct Choice Latency (s)	1.58 ± 0.34	1.66 ± 0.37	1.68 ± 0.51	0.486

## Data Availability

Data will be made available on request.
